# Unmasking Wiskott–Aldrich Syndrome in Adulthood in a Case of Long-Standing Bleeding, Infections, and Steroid-Induced Morbidity

**DOI:** 10.7759/cureus.100461

**Published:** 2025-12-30

**Authors:** Anupam Dutta

**Affiliations:** 1 Department of General Medicine, Assam Medical College & Hospital, Dibrugarh, IND

**Keywords:** eczema, genetic diagnosis, immunodeficiency, thrombocytopenia, wiskott–aldrich syndrome

## Abstract

Wiskott-Aldrich syndrome (WAS) is a rare X-linked primary immunodeficiency characterized by the classic triad of microthrombocytopenia, recurrent infections, and eczema, with most cases diagnosed during infancy or early childhood. Adult presentation is uncommon and often leads to diagnostic delay or misdiagnosis. We report the case of a 28-year-old male who presented with multiple episodes of bleeding, petechiae, recurrent skin and respiratory infections, and chronic eczematous lesions. Over several years, he underwent repeated hospitalizations and was treated for immune thrombocytopenia (ITP) with prolonged courses of oral corticosteroids, resulting in features of iatrogenic Cushing’s syndrome, including moon facies and skin thinning. Despite steroid therapy, his symptoms worsened, and he continued to have intermittent respiratory tract infections, urinary tract infections, and recurrent skin ulcers. The patient’s family history revealed consanguinity and recurrent infections among female relatives, including his mother and maternal aunt, raising suspicion for an inherited immunodeficiency. On examination, he had diffuse eczematous lesions over the hands and feet, petechiae, and Cushingoid features. Laboratory investigations showed persistent thrombocytopenia with a low mean platelet volume, elevated inflammatory markers, and evidence of immune dysregulation. Given the constellation of symptoms, a primary immunodeficiency was suspected, prompting genetic evaluation. Whole-exome sequencing identified a hemizygous pathogenic nonsense variant in the *WAS* gene: NM_000377.3; chrX:48688689; c.961C>T; p.Arg321* in exon 10, confirming the diagnosis of WAS. This variant is predicted to result in the premature truncation of the WAS protein and is consistent with a classical WAS phenotype. This case highlights the challenges in diagnosing WAS in adulthood, especially when initial symptoms mimic more common hematological conditions such as ITP. Early recognition of syndromic features and consideration of inherited immunodeficiencies in persistent thrombocytopenia with eczema can prevent prolonged steroid exposure and facilitate timely, appropriate management.

## Introduction

Wiskott-Aldrich syndrome (WAS) is a rare, inherited, X-linked primary immunodeficiency disorder characterized classically by the triad of microthrombocytopenia, eczema, and recurrent infections. The disorder was first described in 1937 by the German pediatrician Alfred Wiskott, who reported three brothers with bleeding manifestations and recurrent infections who died early in life. Nearly two decades later, Robert Aldrich established the X-linked pattern of inheritance, firmly defining the condition as a distinct genetic syndrome [1,2]. Since then, WAS has served as a paradigm for understanding cytoskeletal regulation in immune cell function.

The estimated incidence of WAS is approximately 1-4 per million live male births worldwide [3]. The disease results from mutations in the *WAS* gene located on chromosome Xp11.23, which encodes the Wiskott-Aldrich syndrome protein (WASP). WASP plays a crucial role in actin cytoskeleton remodeling, which is essential for signal transduction, migration, phagocytosis, and immunologic synapse formation in hematopoietic cells [4]. Deficiency or dysfunction of WASP leads to impaired T-cell, B-cell, natural killer cell, and dendritic cell function, accounting for the combined immunodeficiency state observed in affected patients.

Clinically, patients typically present in early infancy with thrombocytopenia associated with characteristically small platelets, resulting in petechiae, purpura, epistaxis, and gastrointestinal bleeding. Eczematous dermatitis, often severe and persistent, develops early and frequently becomes secondarily infected. Recurrent bacterial, viral, and fungal infections are common due to both cellular and humoral immune defects. In addition, patients with WAS have a marked predisposition to autoimmune manifestations such as autoimmune hemolytic anemia, vasculitis, inflammatory bowel disease, and malignancies, particularly Epstein-Barr virus-associated lymphomas [3,5].

The phenotypic spectrum of *WAS* mutations is broad and ranges from classic WAS to the milder variant of X-linked thrombocytopenia, in which immunodeficiency is minimal. Diagnosis is based on the characteristic clinical features, demonstration of reduced or absent WASP expression by flow cytometry, and confirmatory molecular genetic testing [4,5].

While supportive care with immunoglobulin replacement, antimicrobial prophylaxis, and platelet transfusions remains essential, hematopoietic stem cell transplantation (HSCT) is currently the only established curative therapy and offers excellent long-term survival when performed early in life [6]. Emerging gene therapy approaches have further expanded therapeutic possibilities, making early recognition and diagnosis critically important.

## Case presentation

A 28-year-old male, born out of a consanguineous marriage, presented with a long-standing history of recurrent bleeding episodes, petechiae, repeated infections, and chronic eczema since early childhood. He had multiple episodes of spontaneous mucocutaneous bleeding in the form of petechiae, gum bleeding, and occasional epistaxis, along with recurrent upper and lower respiratory tract infections, repeated urinary tract infections, and recurrent skin ulcers requiring frequent hospitalizations over the last two decades. He also had persistent eczematous lesions involving the hands and feet. There was a significant family history, with his mother and maternal aunt having a history of recurrent skin infections and multiple hospitalizations due to infectious illnesses, suggesting an inherited immunological disorder.

The patient had been evaluated multiple times at different healthcare centers and was repeatedly misdiagnosed as having immune thrombocytopenic purpura (ITP), for which he received several prolonged courses of oral corticosteroid therapy over many years, along with intermittent platelet transfusions during active bleeding episodes, with only transient improvement.

**Figure 1:**
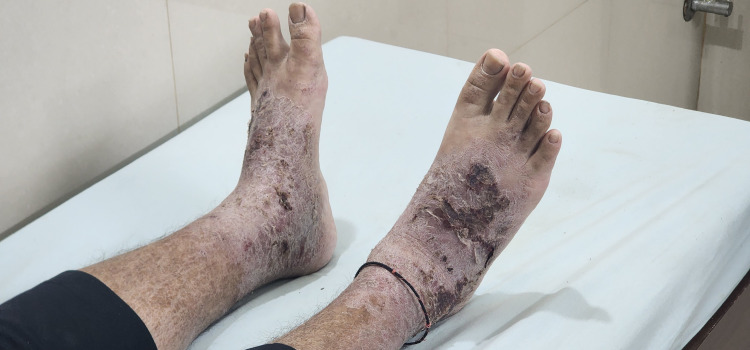
Chronic eczematous lesion in the bilateral dorsum of the foot.

On general physical examination, he was conscious, oriented, and afebrile, with stable vital signs, but had marked Cushingoid features, including moon facies and central weight gain secondary to prolonged steroid exposure, along with generalized pallor, multiple petechiae over the lower limbs, and chronic eczematous lesions with excoriation and hyperpigmentation over the hands and feet (Figure 1). There was no icterus, cyanosis, clubbing, significant lymphadenopathy, or hepatosplenomegaly. Systemic examination revealed bilateral vesicular breath sounds with occasional basal crepitations, normal heart sounds without murmurs, a soft, non-tender abdomen without organomegaly, and no focal neurological deficits.

Laboratory investigations showed persistent thrombocytopenia with platelet counts ranging between 15,000 and 40,000/mm³, with a markedly reduced mean platelet volume consistent with microthrombocytopenia, mild-to-moderate anemia, and a normal to mildly elevated total leukocyte count during infective episodes. The peripheral smear confirmed marked thrombocytopenia with small-sized platelets. The coagulation profile was normal, liver and renal function tests were within normal limits, and serological tests for HIV, hepatitis B, and hepatitis C were negative, while blood cultures during febrile episodes intermittently grew bacterial pathogens. In view of early-onset thrombocytopenia, recurrent severe infections, eczema, poor sustained response to steroids, positive family history, and consanguinity, a primary immunodeficiency was strongly suspected.

Targeted next-generation sequencing revealed a pathogenic nonsense mutation in the *WAS* gene (NM_000377.3) at chromosomal coordinate chrX: 48688689, with a c.961C>T change resulting in p.Arg321* in exon 10, confirming the diagnosis of X-linked WAS. The patient was initiated on intravenous immunoglobulin replacement therapy, antimicrobial prophylaxis, dermatological management for eczema, and a supervised tapering of long-term corticosteroids. He was counseled regarding the definitive role of HSCT and referred to a transplant center for further evaluation, and genetic counseling was advised for the family. This case highlights the significant diagnostic delay and long-term morbidity caused by misdiagnosis of WAS as ITP, emphasizing the importance of recognizing microthrombocytopenia, poor steroid response, recurrent infections, eczema, and family history suggestive of an inherited immunodeficiency.

## Discussion

WAS remains an important but often under-recognized cause of the combined phenotype of microthrombocytopenia, eczema, recurrent infections, autoimmunity, and malignancy in males. Our patient’s long history of bleeding episodes, recurrent respiratory and urinary infections, chronic eczematous dermatitis, microthrombocytopenia, and poor sustained response to corticosteroids, culminating in confirmation of a pathogenic nonsense mutation in WAS (c.961C>T, p.Arg321*), is characteristic of classic WAS. The diagnostic delay in this case, spanning childhood into adulthood with multiple courses of empiric steroid therapy for presumed ITP, highlights common pitfalls in the recognition of inherited platelet/immune disorders and the real-world morbidity of misdirected long-term immunosuppression [7].

In many health systems, WAS continues to be misclassified as refractory ITP, particularly when physicians focus on isolated thrombocytopenia rather than the broader syndromic features. The recognition of microthrombocytopenia (low mean platelet volume) is a crucial clue that should prompt consideration of WAS and other inherited platelet disorders. This morphological distinction was present in our patient and has been emphasized in Indian and international case series [7,8]. The coexistence of eczema and recurrent sinopulmonary and skin infections should raise suspicion for an underlying primary immunodeficiency, especially when steroid therapy provides only transient platelet improvements and leads to Cushingoid complications, as in our patient.

Epidemiologic and cohort data from India have recently broadened the understanding of WAS in South Asia. A multi-institutional Indian cohort described heterogeneous presentations, delayed diagnosis, and limited accessibility to curative therapy, factors that contribute to sustained morbidity and mortality in this region [7]. More recent mutational analyses from Indian cohorts have reported a high proportion of novel WAS mutations and underscored the variability of genotype-phenotype correlations. These data also highlight the complementary diagnostic value of flow cytometric WASP expression testing alongside molecular sequencing in resource-constrained settings [8]. Taken together, these regional studies emphasize the need for clinician awareness, availability of diagnostic testing, and genetic counseling infrastructure.

Long-term complications in untreated or delayed WAS diagnosis include autoimmune disease and malignancy. The association between WAS and lymphoid malignancies is well documented. Several case reports and series from India have described lymphoma complicating WAS, including diffuse large B-cell lymphoma, illustrating the potentially fatal oncologic trajectory if immune dysregulation persists [9]. Autopsy and histopathologic case reports further document the multiorgan consequences of longstanding immune dysregulation in WAS [10]. Rare biologic phenomena such as somatic reversion have also been reported in Indian families and may modulate clinical severity and phenotype, complicating both diagnosis and prognosis [10,11].

Therapeutically, HSCT remains the established curative option for classic WAS. Large registry and Primary Immune Deficiency Treatment Consortium reports demonstrate marked improvements in overall survival and event-free survival over recent decades with modern conditioning and supportive care, with survival approaching rates seen with other inherited immunodeficiencies when transplantation is performed early and with an appropriate donor [12,13]. Indian experience mirrors these global trends but also highlights barriers to timely HSCT access, including delayed referrals, financial and logistic constraints, and donor availability [7,9]. In our patient’s case, late recognition and prior prolonged steroid exposure complicate transplant candidacy and peri-transplant risk, underscoring the advantage of early referral once the diagnosis is suspected.

Gene therapy has emerged as a transformative alternative for patients lacking matched donors or for whom HSCT is high-risk. Long-term results from γ-retroviral and lentiviral vector gene therapy trials have shown durable WASP expression, improved immune function, reduction in bleeding and autoimmunity, and meaningful clinical benefit in most treated patients; however, early γ-retroviral approaches were associated with insertional oncogenesis in some series, prompting the field to transition to engineered lentiviral vectors with improved safety profiles [14]. Recent multicenter gene therapy programs and long-term follow-up reports support gene therapy as an increasingly viable curative approach, and ongoing trials continue to refine vector design and conditioning strategies to maximize benefit and minimize risk [15].

From a practical standpoint, the management of patients with a delayed diagnosis of WAS must include immediate steps to limit further iatrogenic harm and optimize infection control: cessation or rapid tapering of unnecessary long-term corticosteroids when feasible, institution of immunoglobulin replacement for humoral support, antimicrobial prophylaxis tailored to infectious history, and dermatologic care for eczema. Platelet transfusions should be used judiciously for active bleeding, and live vaccines should be avoided in those with significant immunodeficiency. Genetic counseling for the family is essential, particularly given the X-linked inheritance pattern and reports of symptomatic or carrier-manifesting females related to skewed X-inactivation and somatic events [16].

This case also underlines system-level priorities. First, improved clinician education about inherited thrombocytopenias and syndromic causes of thrombocytopenia is needed to reduce misdiagnosis. Second, strengthening laboratory capacity for platelet indices, WASP flow cytometry, and molecular testing will shorten diagnostic latency, an imperative documented in Indian cohort analyses [7,10]. Third, enhancing access to curative therapies through policy initiatives, funding pathways, and transplant/gene therapy collaborations can convert molecular diagnosis into life-saving treatment [11,12]. Finally, publication of regional outcomes and case reports, both historic and recent, adds to collective experience and aids clinicians in recognizing the diverse presentations of WAS across different populations [9,8,16].

## Conclusions

This patient’s course typifies how WAS may be masked as refractory ITP, with substantial morbidity from both disease and inappropriate therapy. Early suspicion prompted by microthrombocytopenia, eczema, and recurrent infections should trigger targeted testing (WASP flow cytometry and *WAS* gene sequencing) and timely referral for curative therapy. The expanding Indian literature and global advances in HSCT and gene therapy provide a compelling rationale for earlier recognition, rapid diagnostic workup, and definitive treatment planning to prevent the long-term complications exemplified in this case.

## References

[1] Thrasher AJ, Kinnon C (2000). The Wiskott-Aldrich syndrome. Clin Exp Immunol.

[2] Aldrich RA, Steinberg AG, Campbell DC (1954). Pedigree demonstrating a sex-linked recessive condition characterized by draining ears, eczematoid dermatitis and bloody diarrhea. Pediatrics.

[3] Ochs HD, Thrasher AJ (2006). The Wiskott-Aldrich syndrome. J Allergy Clin Immunol.

[4] Notarangelo LD, Miao CH, Ochs HD (2008). Wiskott-Aldrich syndrome. Curr Opin Hematol.

[5] Sullivan KE, Mullen CA, Blaese RM, Winkelstein JA (1994). A multiinstitutional survey of the Wiskott-Aldrich syndrome. J Pediatr.

[6] Moratto D, Giliani S, Bonfim C (2011). Long-term outcome and lineage-specific chimerism in 194 patients with Wiskott-Aldrich syndrome treated by hematopoietic cell transplantation in the period 1980-2009: an international collaborative study. Blood.

[7] Suri D, Rikhi R, Jindal AK (2021). Wiskott Aldrich syndrome: a multi-institutional experience from India. Front Immunol.

[8] Gaikwad P, Bargir UA, Jodhawat N (2024). Mutational landscape of patients with Wiskott Aldrich syndrome: update from India. J Clin Immunol.

[9] Senapati J, Devasia AJ, David S, Manipadam MT, Nair S, Jayandharan GR, George B (2014). Diffuse large B cell lymphoma in wiskott-Aldrich syndrome: a case report and review of literature. Indian J Hematol Blood Transfus.

[10] Rikhi R, Basu S, Arora K (2024). Somatic reversion in Wiskott-Aldrich syndrome: case reports and mechanistic insights. Scand J Immunol.

[11] Patil MG, Verma S, Avuthu OP, Subramanian K, Tambolkar S, Mane SV (2024). Wiskott-Aldrich syndrome: a report of a rare X-linked disorder. Cureus.

[12] Ferrua F, Cicalese MP, Galimberti S (2019). Lentiviral haemopoietic stem/progenitor cell gene therapy for treatment of Wiskott-Aldrich syndrome: interim results of a non-randomised, open-label, phase 1/2 clinical study. Lancet Haematol.

[13] Burroughs LM, Petrovic A, Brazauskas R (2020). Excellent outcomes following hematopoietic cell transplantation for Wiskott-Aldrich syndrome: a PIDTC report. Blood.

[14] Braun CJ, Boztug K, Paruzynski A (2014). Gene therapy for Wiskott-Aldrich syndrome--long-term efficacy and genotoxicity. Sci Transl Med.

[15] Moratto D, Giliani S, Bonfim C (2011). Long-term outcome and lineage-specific chimerism in 194 patients with Wiskott-Aldrich syndrome treated by hematopoietic cell transplantation in the period 1980-2009: an international collaborative study. Blood.

[16] Gupta MC, Agarwal VK, Mittal AK, Rajvanshi VS (1964). Wiskott-Aldrich syndrome. A case report. J Assoc Physicians India.

